# Electrical Conductivity and Optical Properties of Pulsed Laser Deposited LaNi_5_ Nanoscale Films

**DOI:** 10.3390/ma11081475

**Published:** 2018-08-19

**Authors:** Daniela Todoran, Radu Todoran, Zsolt Szakács, Eugen Anitas

**Affiliations:** 1 Department of Economics and Physics, Technical University of Cluj Napoca, North University Centre of Baia Mare, 430083 Baia Mare, Maramures, Romania; todorandaniela05@yahoo.com (D.T.); todoran_radu@yahoo.com (R.T.); 2 Bogoliubov Laboratory of Theoretical Physics, Joint Institute for Nuclear Research, 141980 Dubna, Moscow Oblast, Russia; anitas@theor.jinr.ru; 3 Horia Hulubei National Institute of Physics and Nuclear Engineering, 077125 Bucharest-Magurele, Romania

**Keywords:** nanoscale films, LaNi_5_ intermetallic alloy, pulsed laser deposition, electrical and optical properties, temperature dependence, scale dependentce

## Abstract

This work presents pulsed laser deposition as a method to obtain unoxidized LaNi_5_ nanoscale films and describes their temperature and thickness dependent electrical conductivity and the spectral dispersions of some optical properties. AB_5_-type rare earth element (REE)-nickel compounds are currently studied from both theoretical and practical points of view. Special challenges are posed during the preparation of these nanomaterials, which can be overcome using finely tuned parameters in a preparation process that always involves the use of high energies. Film deposition was made by laser—induced vaporization, with short and modulated impulses and electro–optical tuning of the quality factor, mainly on glass and one SiO_2_ substrate. Deposition geometry dependent linear thickness increase, between 1.5–2.5 nm per laser burst, was achieved. Film structures and phase compositions were determined using XRD and discussed in comparison with films obtained by similar deposition procedures. Temperature and scale dependent properties were determined by studying electrical conductivity and optical properties. Electrical conductivity was measured using the four-probe method. The observed semiconductor-like conductivity for film thicknesses up to 110 nm can be explained by thermal activation of electrons followed by inter-insular hopping or quantum tunneling, which, on the other hand, modulates the material’s native metallic conductance. Films with thicknesses above this value can be considered essentially metallic and bulk-like. The spectral behaviors of the refractive index and absorption coefficient were deduced from differential reflectance spectroscopy data acquired on a broad ultraviolet, visible, near- and mid-infrared (UV-VIS-NIR-MIR) domain, processed using the Kramers-Krönig formalism. Their study led to the identification of the allowed interband transitions. Electronic behavior in the energy bands near the Fermi level and in the surface and interface-states was described, discussing the differences between experimental data and the classical free-electron theoretical model applied for the bulk intermetallic alloy, in correlation with theoretical optical properties or experimental X-ray photoelectron spectroscopy (XPS) results from references. However, the dielectric-like shape of the reflectance of the thinnest film was in accordance with the Lorentz–Drude model.

## 1. Introduction

The importance of rare earth element (REE) compounds is undeniable in modern technologies, proven by the number of studies published for both their theoretical description and practical applications. Lanthanum is the prototype of its group of transition metals. Its compounds with cations like Boron are semiconductors; as such, they can be used to construct thin film transistors [1], or as a plasmonic material when partially replaced by Eu, because of wide band near-infrared (NIR) absorption properties [2]. Linked to organic molecules lanthanum can produce super-hydrophobic surfaces [3] or act as a catalyst in low polydispersity polymerization processes [4]. When alloyed with Mg, or in higher Mg alloys, it displays remarkable strengthening properties and activates additional deformation modes [5]. Lanthanum can also influence the microstructure of steel [6]. Probably, its best known application is hydrogen storage in its AB_5_-type alloy with Ni. Early studies of RNi_5_ type compounds—where R is a REE like La or Gd, and where Ni can be replaced in a certain amount by some other metal—were made to link theoretically computed electron energy band structures to the observed calorimetric [7,8] and magnetic properties [9,10,11]. Lanthanum’s hydrogen storage potential was also recognized early [12]. Nowadays, most of the Ni-metal hydride batteries use this type of compound in the cathode, while cutting-edge research methods are applied to enhance storage capacity. This was done, for example, by constructing Mg-containing superlattices or hybrid structures [13,14,15] to increase the speed and the number of reversible hydrogen sorption cycles by correlating experimental data with ab-initio calculations [16], or by optimizing functioning conditions [17]. The effect of the magnetic field on thermal conduction and sorption properties of the La-Ni-Mg hydrogen storage beds was also studied [18]. The fractal dynamics of the deuterium sorption process on this fractal structured surface was determined using small angle neutron scattering (SANS) [19]. Some less common applications of the lanthanum compound’s hydrogen sorption properties were also proposed, such as for a catalyst during the production of synthetic natural gas [20] or as an actuator for an artificial muscle [21].

Such a wide REE demand and limited availability has implications from environmental, sustainable economic development, and public health points of view, influencing both social and governmental behaviors. A review of global REE abundance and current mining and recovery methods, underlining environmental costs, is presented in [22], while evaluation of global REE demand is considered in [23]. China’s dynamic computable equilibrium supply model forecast up to 2025 was also described [24], together with its sustainable development policies [25]. Reduction of environmental burdens and a European production chain was also proposed [26]. Social claims and impact related to environmental concerns of RRE-mining were analyzed, and development of mediated intergovernmental closed-loop policies were proposed as a solution to reenter REE in the Global Value Chain from end-of-life products through industries based on ecological principles [27,28]. The feasibility of a recycling strategy of ultra-fine REE powders based on two-liquid flotation was also reviewed [29]. Impact of REE industrial pollution was evaluated by direct quantification [30] and from a radiological point of view [31], and sorption using natural sorbents was proposed for pollution remediation [32].

An alternative method for the reduction of REE usage, while also modulating the obtained compound’s surface, porosity, and structure, together with its conductance and optical properties, is resorting to nanomaterials. Obtaining nanoscale REE-Ni compounds poses special technical challenges and requires high energy deposition methods and a careful fine tuning of the parameters used during the production process [33,34,35,36]. The first technical challenge that must be surpassed is linked to the fact that high energies or temperatures of at least 1000 K are needed. In recent studies, highly disperse LaNi_5_ nanoparticles, in the domain of 100–350 nm, with a statistical mode around 200–250 nm, were obtained using combustion-reduction, and precipitation-reduction methods without the use of a substrate [33]. Recognizing the need for a suitable substrate led to a decrease in size and dispersity, to around 5–50 nm, with a statistical mode around 15–20 nm [34]. Our team previously also reported successful preparation of CeNi_5_ nanoscale films on a glass or SiO_2_ substrate using high energy or temperature-deposition techniques [35,36]. At that time, our first method of choice was resistive heating vaporization of the bulk powdered CeNi_5_ alloy, placed on a W filament. This led to partial dissociation of Ni from of the initial compound, as was proven by the obtained XRD patterns, together with smaller than 3% deposition efficiency, without any control on the deposited nanomaterial’s size. The poor results were due to the high difference between the two metal’s boiling temperatures, the technical limit of the W filament, which functioned just below its melting point, and the filament’s thermal inertia. Therefore, this method had to be discarded. The second method applied to produce nanoscale films was laser induced vaporization in a low pressure Ar filled chamber, using a 30 MW pulses from a ruby laser, with short and modulated impulses. This lead to the full control of the layer-by-layer film growth, with a couple of nanometer thickness increase per laser impulse, depending on the deposition geometry. Because good results were obtained this way, we also applied this deposition method and these substrates in the preparation of the presently described LaNi_5_ nanometer thickness films. For a comparison, we should refer to the work of Gondoni et al. where transparent conductive oxide films were obtained using the same deposition technique, with working parameters that are comparable, in some cases, with ours [37,38].

This work is a study dedicated to the optical and electrical properties of nanometer thickness LaNi_5_ films, deposited by laser induced vaporization on glass or SiO_2_ substrates. A very good linear layer thickness increase vs. number of laser impulses, determined using interferometric optical microscopy, was observed over the whole laser impulse count domain. This way, the films used in our study were determined to have the following thicknesses: 4.0 nm, 4.5 nm, 22 nm, 38 nm, 42 nm, 68 nm, 110 nm, and 180 nm on glass substrate, and 36 nm on SiO_2_ substrate. The influence of the gas pressure on the structure of the deposited films will be discussed according to the findings from referenced materials [37,38].

Temperature dependent electrical conductivity measurements, made for different layer thicknesses, exhibited semiconductor-like behaviors below 110 nm thickness, up to temperatures around 0 °C, and metallic behavior for the 180 nm thick film for any temperature above 80 K. For the 110 nm thick film, the semiconductor-like temperature dependence of conductance gets compensated for by the metallic behavior, leading to an approximately constant conductance value over a wide range of temperatures. The semiconductor-like electrical conductivity features were due to the insular film deposition patterns and could be better observed in the behavior for the 68 nm film. Inter-insular hopping was identified as the source of an energetic feature which gave a band gap-like feature. The thickest studied film of 180 nm exhibited only bulk-like metallic electrical properties and was considered to be continuous.

Film thickness-dependent absolute reflectance and transmittance values, at the wavelength of a HeNe laser, with substrate influence removed using a single-beam differential technique, revealed that bulk-like LaNi_5_ optical properties are regained at thicknesses just above approximately 68 nm. The film at this thickness had bulk-like optical transmittance and reflectance values, for the HeNe laser wavelength of 632.8 nm, but semiconductor-like electrical conductivity, due to its insular structure.

The electron energy spectrum in metal alloys strongly influences their optical properties and can be studied when most of the free electrons are bound in the surface states or captured at the interfaces. Therefore, relative differential reflectance spectroscopy measurements were made for films with different thicknesses in the 0.185–25 μm spectral domain, leading to the identification of the allowed electronic interband transitions [39]. These results are presented and discussed in this work only for the most important UV-VIS-NIR domain and for the films up to 42 nm, where the effects were most noticeable. The relative spectral dependencies were calibrated using the absolute differential reflectance and transmittance measurements obtained using a HeNe laser. After that, the absolute differential reflectance spectroscopy data was processed using the Kramers-Krönig formalism. This led to the discussion of the spectral behavior of the refractive index *n* and extinction coefficient *k*. Comparison of the obtained results with the classical Drude-Sommerfeld free electron model and the Lorentz-Drude model for a dielectric, alongside with their correlation with X-ray photoelectron spectroscopy (XPS) measurements [40,41,42] and theoretically computed optical properties of the bulk [41,42,43], elucidated the photon-electron interaction mechanisms and allowed us to propose suitable models that can be applied to describe the films with different thickness. The thinnest films, up to a couple of tens of nanometer thickness, displayed optical and electrical properties characteristic of insularly deposited materials, and received most of the attention. Another conclusion that could be drawn is that the initial isles gradually merged later during the deposition process. The screening effect of the free electrons on the spectral behavior of the considered optical properties was heavily felt right from 38 nm thickness.

## 2. Materials and Methods

The bulk LaNi_5_ was obtained from 99.9% pure La and Ni metals, in stoichiometric proportions, using the classical arc electrode melting method in a low-pressure Ar chamber. The alloy was milled in an agate mortar. Approximately 10 mg powder, which passed through a 1200 mesh sieve, was placed on a Cu sample holder and cooling system, in a 10^–5^ Torr pressure, Ar filled deposition chamber.

Mainly glass, and a single SiO_2_ substrate was used. These substrates were cut to such a size that they would fit the spectrometers’ sample chamber. They were cleaned using doubly distilled water and isopropyl-alcohol and ablated by accelerated Ar ions. For each substrate, reflectance and transmittance measurements were made for calibration purposes, right after their Ar ion ablation and just before starting the deposition process. Absolute reflectance and transmittance measurements were made using a HeNe laser (LGN-215, URSS) system’s 632.8 nm wavelength, calibrated using an Ag mirror (R > 0.94 ± 0.0005). A vertical incident angle with a deviation smaller than 1.5 arcmin was fixed using a goniometer. The relative single-beam reflectance spectroscopy measurements were recorded using the Specord (DDR)—M40 and 75IR—spectrometers for the two separate domains of 185–950 nm and 2.5–25 μm, respectively.

**Figure 1:**
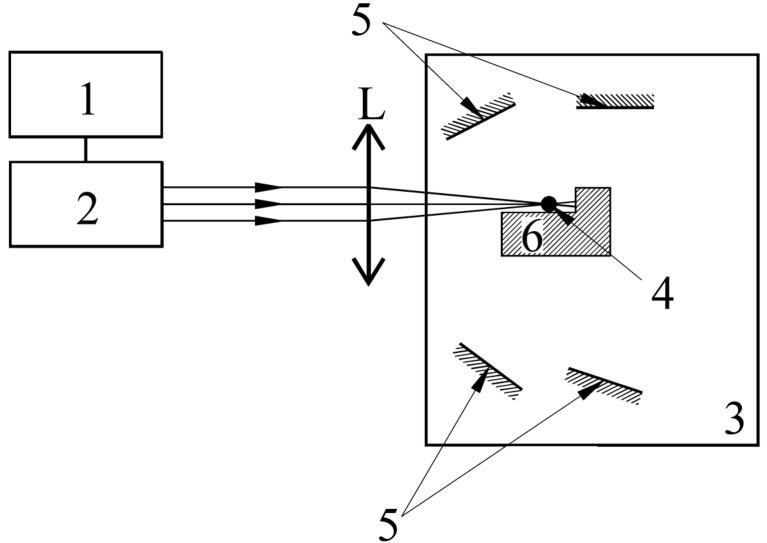
Schematic representation of the pulsed laser induced vaporization system [36]. 1: electro-optical system to tune the laser’s quality factor, 2: laser resonator chamber, L: laser radiation focusing lens system, 3: low pressure Ar filled deposition chamber, 4: bulk sample, 5: deposition substrates, 6: bulk sample holder and cooling system.

The substrates were positioned in the deposition chamber, around the sample holder at distances of 3–5 cm, and at incident angles deviated from perpendicular by 10–15°, to obtain the different deposition thicknesses. The schematic representation of the used deposition setup is presented in Figure 1 [36]. The Al_2_O_3_(Nd^3+^) ruby laser (experimental LGIN-503, URSS) was used at a power output of 30 MW, with a pulse length of 20 ns and a pulse frequency of 10 Hz. The laser beam focus spot had a diameter of approximately 100 μm. The laser’s fundamental (1064 nm) and second harmonic (532 nm)—the one which was also used to observe the bulk sample—wavelengths were selected for energy delivery by filtering. The Cu radiator and these energy settings allowed for the achievement of instant vaporization and kept sufficient bulk compound in a liquid state at all times up to the end of the deposition process.

Film thickness measurements were made using an interferometric optical microscope (LOMO MII-5, URSS) and a Na lamp’s 588.995 nm wavelength [36]. This method also allowed for the observation of the insular films deposition patterns, when the objective’s resolution was suitable. Because of the limitation of the applied method, only thicknesses starting from just above 40 nm could be measured, with a precision of 10%. Lower thicknesses were determined by extrapolation, correlating the number of the laser impulses with measured thicknesses in the already known cases.

Phase composition of the deposited films was determined by comparing the X-ray diffractograms (DRON-8, Russia), obtained using the CuK_α_ radiation, with those of the bulk, or bibliographic references. Nanoscale films always exhibit broadened peaks because of small crystallite sizes and the presence of microstrain distorting the crystal structure. These quantities were evaluated using Williamson-Hall analysis [44]. Broadening is amplified by the instrumental factor. Therefore, we had to previously calibrate the instrument by determining the corrections for the crystalline silicon powder’s diffraction peaks, also considering the effect of CuK_α2_ radiation. After correcting the obtained diffractograms of our thin films for instrumental effects, the Williamson-Hall equation could be considered:

(1) $$\alpha \cos\theta =\frac{0.9\lambda }{D}+4\varepsilon \;\sin\theta$$

where *α* is the remaining peak broadening, *θ* the diffraction angle, 0.9 is a shape factor, *D* the crystallite size, and *ε* the microstrain. A linear fit of the data in the (*α*cos*θ*, 4sin*θ*) plane has the intercept proportional with the inverse of the crystallite size and the slope equal with the microstrain. In our case, uniform deformations were considered.

All electrical conductivity and optical measurements that followed were performed after a single initial film surface cleaning, using accelerated Ar ions, at a low potential. This way, the possible contamination and oxidation from just the first couple of deposited atomic layers should have been removed. Electrical conductivity was measured by the classical four-probe method [45], in the temperature interval of 80–300 K.

The same measurement procedure, at the HeNe laser’s wavelength, that was used for the substrates allowed for the determination of the deposited films’ absolute differential reflectance and transmittance values, without the substrate’s contribution. After this, relative differential single-beam reflectance spectroscopy determinations were performed on the films, using the same procedure and apparatus as for the substrates. So that, finally, absolute reflectance spectral dispersion values were obtained for each film, without the substrates’ contribution. To cover a spectral domain as wide as possible, the experimental data were extrapolated down to 125 nm using an 1/*a*^ν^–type law, where ν is the photon frequency, and the dimensionless coefficient *a* was determined using two lines from the Lyman series of a deuterium lamp and a LiF window. These data were processed to compute the refractive index *n* and the extinction coefficient *k* using the Kramers-Krönig formalism [46,47]. Variations of the above-mentioned method were previously applied by us and gave good results on natural mineral semiconductors [48], solid–liquid interfaces [49,50], and other intermetallic nanoscale films [35,36].

Optical properties were compared with the appropriate Drude model. We derived our conclusions for the films with thicknesses up to a couple of tens of nanometers based on the differences observed between the behavior of our deposited films and the bulk material described by the Drude-Sommerfeld free electron model [51]. The assumption on bulk material’s metallic behavior was supported by the previous valence band conclusions from the work of Burzo et al. [41,42]. In this case, the complex dielectric function’s real part *ε*_1_—the dielectric constant or dielectric permittivity—and imaginary part *ε*_2_—the dielectric loss function—can be approximated, for *ω* < *Γ*, as:

(2) $$\varepsilon_{1}\left(\omega \right)\approx \varepsilon_{\infty }-{\left(\frac{\omega_{p}}{\omega }\right)}^{2},$$

(3) $$\varepsilon_{2}\left(\omega \right)\approx \Gamma \frac{\omega_{p}^{2}}{\omega^{3}},$$

so that the refractive index *n* and the extinction coefficient *k* can be computed using:

(4) $$2n{\left(\omega \right)}^{2}=\sqrt{\varepsilon_{1}^{2}\left(\omega \right)+\varepsilon_{2}^{2}\left(\omega \right)}+\varepsilon_{1}\left(\omega \right),$$

(5) $$2k{\left(\omega \right)}^{2}=\sqrt{\varepsilon_{1}^{2}\left(\omega \right)+\varepsilon_{2}^{2}\left(\omega \right)}-\varepsilon_{1}\left(\omega \right).$$

The reflectivity can be computed using:

(6) $$R\left(\omega \right)=\frac{{\left[n\left(\omega \right)-1\right]}^{2}+k^{2}\left(\omega \right)}{{\left[n\left(\omega \right)+1\right]}^{2}+k^{2}\left(\omega \right)}$$

The values for the electron plasma energy, *ω_p_*, and the parameter that accounts for the net contribution of the positive ion cores, *ε*_∞_, were taken to be equal with the ones determined in the work of Chen et al. [43]. The constant relaxation rate, *Γ*, was the only parameter to be fitted. This procedure gave the best approximation for the behavior of the 42 nm film that already exhibited mainly bulk-like optical properties. Fitting was done only on the narrower energy domain of 1.5–4.5 eV to minimize the frequency dependence of the relaxation parameter, which can be due to electron-electron interactions. On the other hand, to describe the dielectric-like behavior of the thinnest film’s reflectance, we approximated its plateau-like shape using the Lorenz-Drude model for dielectrics. This model was also used to discuss the shape of its extinction coefficient.

## 3. Results and Discussions

### 3.1. Film Thickness and Phase Composition

**Figure 2:**
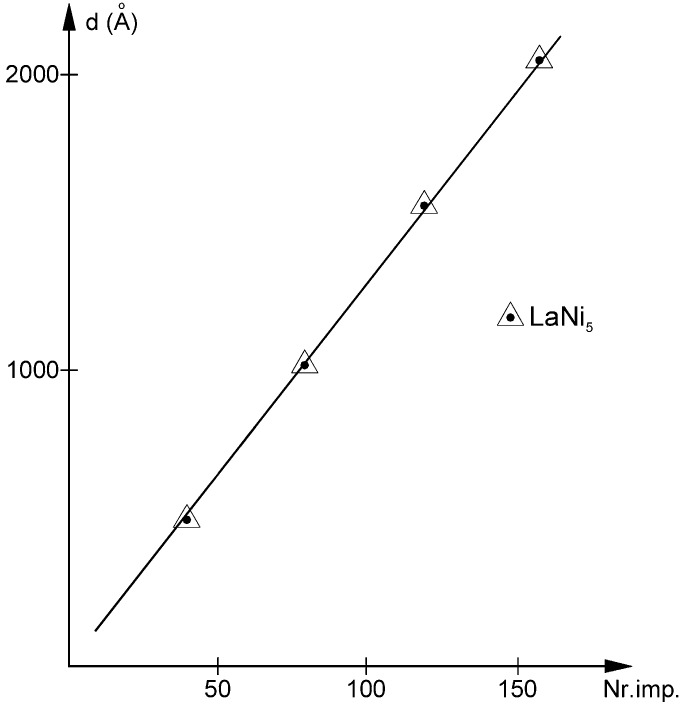
Dependence of the LaNi_5_ film thickness vs. laser impulses number.

The graphical representation of film thickness measurements made using interferometric optical microscopy shows a very good linear thickness increase vs., laser impulse, as can be seen in Figure 2. This allowed us to set the intercept in the graph’s origin and determine the layer thickness increase per laser impulse to be between 1.5–2.5 nm for the differently positioned samples. An interesting point that should be noted is that the same layer thickness increase was obtained for the CeNi_5_ nanoscale films [35,36].

This good linearity also permitted the extrapolation and calculation of film thicknesses below the domain of the interferometric microscope *d* < 40 nm from the number of laser impulses. This film thickness domain is usually measured using ellipsometry. However, this technique requires that the spectral dispersion of the optical functions *k*—the extinction coefficient—and *n*—the refractive index—or *ε*_1_—the dielectric constant or dielectric permittivity—and *ε*_2_—the dielectric loss function—to be known “a priori”. This, in turn, needed the film thickness measurements, and their determination was done later in our study. It is also true that the main impediment in using ellipsometry on metallic films remains the small difference between the intensities of the ordinary and extraordinary rays, not like for dielectrics or semiconductors.

There was an insular deposition pattern that was observed in the case of the 40–68 nm LaNi_5_ films using interferometric optical microscopy. It is comparable with the SEM images obtained, for example, in the works of Gondoni et al. [37,38]. In that instance, SEM imaging was used to correlate the structure of pulsed laser deposited doped semiconductor transparent conductive oxide thin films with their optical and electrical properties. Insular deposition patterns, for the first few tens of nanometers of film, and insight in the porosity of the films were easily gained this way. The films deposited in that study were proposed to replace another expensive raw material, indium, used in transparent conductive films such as indium–tin oxide (ITO) with the cheaper and more environmental friendly Al–doped ZnO. The study presented in this work resembles the sample preparation conditions of those in the work of Gondoni et al. [37], with a gas pressure in the deposition chamber of 0.01 Pa. In that situation, the SEM images clearly show insular growth patterns for the first few tens of nanometers. Because low deposition-chamber gas pressure leads only to a few collisions between the material ejected by the laser pulse and the gas molecules, the mean free path in the plume can be considered a long straight line all the way to the deposition substrate, so that a compact nanoscale film can be obtained. Increasing the gas pressure leads to a significant increase in the number of collisions and possible capture of filler gas atoms, shortening the mean free path and making it more sinuous. This completely changed the structure and properties of the deposited films, making them resemble the fractal structures obtained during diffusion limited aggregation (DLA) processes [52]. In this later case, high porosity and specific surface materials were obtained [38]. For the LaNi_5_ films, the increased porosity and specific surface could be applied, for example, to enhance hydrogen sorption properties. However, this research needs to be completed with specific studies [19,53].

**Figure 3:**
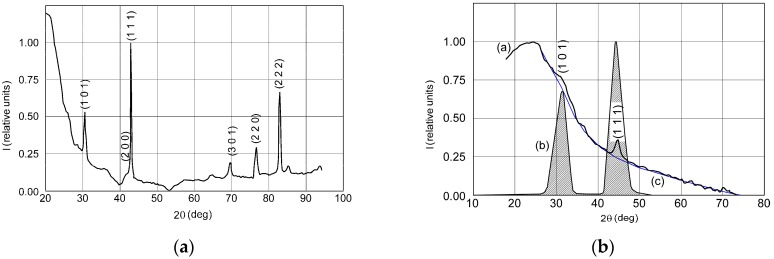

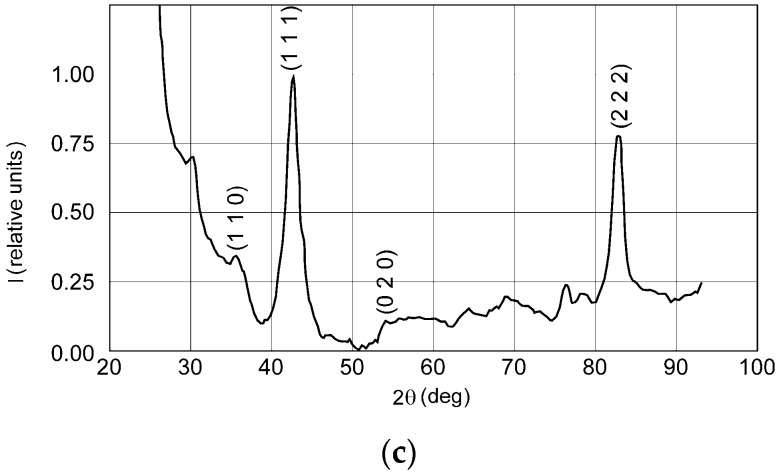
XRD patterns of: (**a**) bulk LaNi_5_ powder; (**b**) 4.0 nm thick film; (**c**) 68 nm thick film.

The XRD patterns of the 4.0 nm and 68 nm thick films—Figure 3b,c—display polycrystalline films with no supplementary phases due to dissociated Ni, when compared to diffractograms from the bulk LaNi_5_—Figure 3a—or with those found in the references [33,34]. In all cases, substantial peak-broadening and substrate scattering effects can be observed, which are well-known characteristics for nanoscale films. However, even for the thinnest film, the reflections from the (101), (110), (111), (020), and (301) family of crystallographic planes are obvious. For the 68 nm thick film we obtained a crystallite size of 17 nm, while the micro-strain was estimated to be under 0.5%. This way, one can conclude that the CaCu_5_ type structure of the deposited LaNi_5_ films is essentially preserved.

### 3.2. Electrical Conductivity

**Figure 4:**
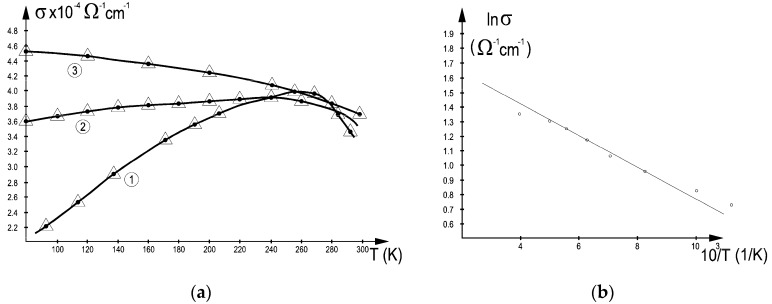
(**a**) Temperature dependent electrical conductivity for the LaNi_5_ films of thickness (1) 68 nm (2) 110 nm and (3) 180 nm. The thick line is a guide for the eye, (**b**) Richardson plot of conductivity σ_(1)_ from (**a**), curve (1).

The behavior of the electrical conductivity determined for the 68 nm, 110 nm, and 180 nm thick films is represented in Figure 4a, curves (1)–(3), in this order. 

For the 68 nm film, an increase in conductivity with temperature up to around 0 °C is observed. This temperature-dependent variation becomes minimal on a large temperature domain for the film with 110 nm thickness. These are evident semiconductor-like variations. Above this thickness, for the 180 nm film, a continuously decreasing tendency is present. The continuous decrease in electrical conductivity with increasing temperature, from Figure 4a curve (3), is characteristic to metals, in which case electrical resistance increases with temperature. This way, one concludes that the 180 nm film displays mainly bulk-like metallic properties. This is also supported by the optical reflectance and transmittance measurements that will be discussed later for the films above 68 nm thickness.

Probably the most interesting behavior is the one from Figure 4a curve (1). When it is displayed as a Richardson plot, which is characteristic to the study of semiconductors—Figure 4b—one observes a good linearity up to the conductivity maximum that is reached at a temperature around 260 K. The activation energy that can be computed using the intercept from Figure 4b, using only five points from the center, and having the highest coefficient of determination, with a value equal to 0.9953 and *p* < 0.00013 is of 5.1 ± 0.3 meV. The second-best linear regression is given by the case when all points from the determined behavior are considered, with just a little bit lower coefficient of determination, with a value of 0.9915 and, of course, with a better p value of 2 × 10^–7^. In this case, the band gap has an approximately 10% lower value of 4.5 ± 0.25 meV. This behavior is different from the one found in the case of our deposited CeNi_5_ where two such activation energies were identified but where one of these energies had a just slightly different value of 4 meV [35,36].

For the interpretation of the semiconductor-like conductance of the metallic films on insulator substrates, one can resort to the insular deposition model. This was also proven to be the case for our deposited CeNi_5_ films [35,36]. The determined activation energy can be interpreted as the inter-insular hopping energy of the electrons originating from the metal alloys surface states generated by the thermoelectronic effect. After the electron extraction process is finished, these electrons can hop to the neighboring islands due to their remaining thermal energy or because of quantum tunneling, so that electrical conduction occurs. This is also enhanced by the number of surface states present in the discontinuous insular films, which is very high when compared to that of the bulk metal. In addition, because the free electrons are captured in these surface states, the level occupancy number near the Fermi energy and the optical effects due to the free electrons decrease, so that the number of possible electronic transitions increases, opening the way to probe this energy region by optical means.

An intermediate behavior between metallic and semiconductor-like, with a nearly constant conductivity over the studied temperature domain, can be observed in Figure 4a curve (2) for the 110 nm film. Here, the two conduction processes tend to equalize their effects over a wide temperature range. In one case, this is due to the decrease in inter-insular hopping distances, so that smaller thermal energy is needed to hop between isles, and in the other, it is characteristic of metallic conduction, which decreases with increasing temperature. This way, a nearly constant conductance was obtained up to nearly room temperature.

These two different conduction behaviors can no longer be observed above approximately 0 °C, where the deposited film exhibits metallic properties, with conductivity constantly decreasing with increasing temperature.

### 3.3. Optical Reflectance and Transmittance

**Figure 5:**
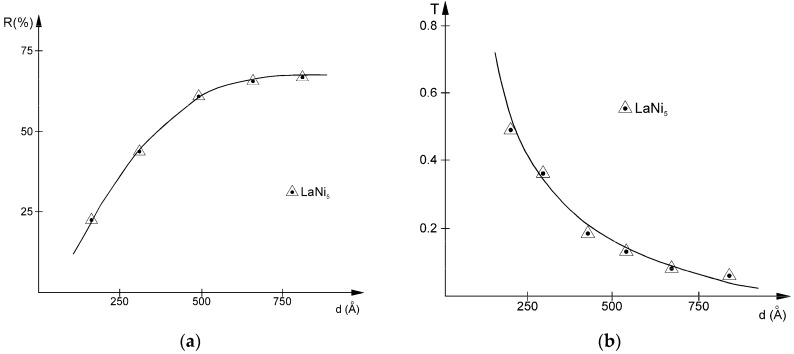
Film thickness dependence of (**a**) absolute differential optical reflectance and (**b**) transmittance for λ_HeNe_ = 632.8 nm.

The variation of the absolute differential optical reflectance and transmittance with film thickness, for the films deposited on glass substrate, obtained using the HeNe laser’s 632.8 nm wavelength, can be seen in Figure 5a,b, respectively. As mentioned before, a single-beam differential technique was applied to remove the substrate’s effect.

These measurements were made not just to calibrate further spectroscopic determinations. An important observation that can be made using them is that the curves tend to level out at film thicknesses above approximately 68 nm. The reflectance value at this thickness is 67%, compared to that of the bulk alloy, which was determined to be equal with 68%, so that this film already exhibits bulk-like optical properties for this wavelength. The measured reflectance values are approximately 5% higher than those obtained for our CeNi_5_ films [35,36], while transmittance values tend to be equal with those of the cerium compound. The classical analytical relation linking *T* and *R* which fits these figures and considers the optical absorption coefficient *α* and film thickness *d* is:

(7) $$T=\frac{{\left(1-R\right)}^{2}e^{-\alpha d}}{1-R^{2}e^{-\alpha d}}$$

Because these nanoscale films are insular in shape, for the main part of the thicknesses considered in this study, the substrate’s reflectivity must be removed. This is done using single-beam differential reflectance spectroscopy measurements [39]. Small isles deposited for low thickness films increase in area as thickness increases and an ever-smaller substrate area remains uncovered, until a continuous film with metallic properties is obtained, at around 180 nm, as is deduced from the conductivity data. However, light can probe a couple of tens of nanometers of nontransparent solid film. Therefore, it is important this method is also applied for the thicker nanoscale films, to compensate for the uncovered substrate’s signal.

The spectra obtained this way contains information about the electron energy bands between which optical transitions are allowed, directly determinable by the position of the particularities that are identifiable in the measured data as maxima or inflection points. Consequently, it cannot characterize alone the whole electron energy spectrum near the Fermi energy. Therefore, optical studies must be complemented by comparison with XPS measurements. In our case, this was done using bibliographic material. However, optical probing has an advantage in penetration depth, which is around an order of magnitude higher than that of XPS. An XPS study of this compound’s thin films can be found in the work of Skoryna et al. [40]. Correlated studies of magnetic properties with electronic ones obtained from XPS valencene band measurements and the behavior of the calculated density of states (DOS), can be found in the works of Burzo et al. [41,42]. Computed spectral behaviors of some optical functions for the bulk alloy, using the planewave pseudo-potential method, based on density functional theory, can be found in the work of Chen et al. [43]. All these data were used for comparison with our determinations.

**Figure 6:**
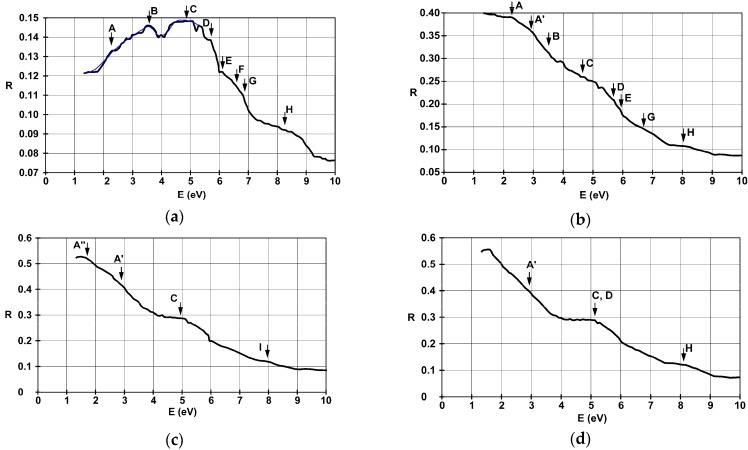

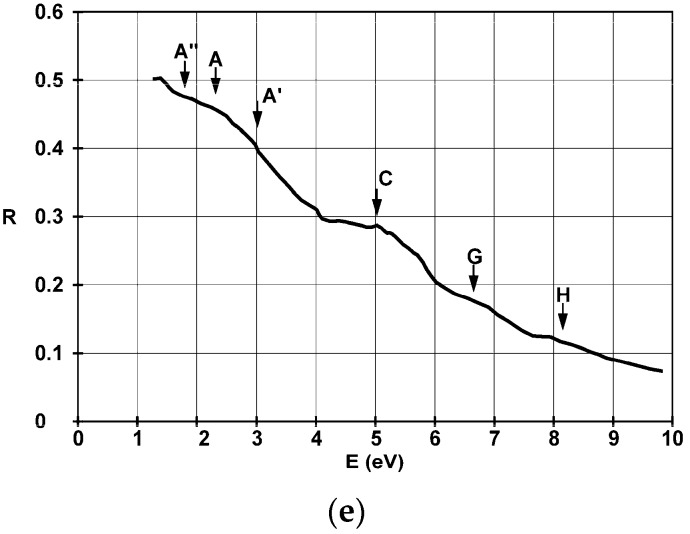
Spectral dispersion of reflectance, with substrate contribution removed, for the films deposited on glass substrate with thicknesses of (**a**) 4.5 nm, (**b**) 22 nm, (**c**) 38 nm, (**d**) 42 nm, and (**e**) 36 nm on SiO_2_. The blue curve in part (**a**) represents the computed behavior using the Lorenz-Drude model for a dielectric.

The spectral dispersions of the calibrated and rescaled optical reflectivity, using absolute reflectance data, covering just the most important UV-VIS-NIR domains, are represented in Figure 6a–e for the films deposited on glass substrate with thicknesses of 4.5 nm, 22 nm, 38 nm, and 42 nm, and on SiO_2_ substrate with a thickness of 36 nm.

The observed allowed interband transitions are summarized in Table 1.

A good concordance is shown by our spectral reflectance data with previous XPS studies from the work of Skoryna et al. [40] and the measured XPS valence band spectra and computed convoluted DOS from the works of Burzo et al. [41,42]. The same good correlation between direct energy levels probing near the Fermi energy using XPS and optical reflectance data describing possible transitions near it was obtained in our previous work on CeNi_5_ nanoscale films [35].

Turning our attention to the particularities found in Figure 6a, in the case of the thinnest film that was studied, one can identify three broad transitions with energies of 8.2 eV, 6.5 eV, and 4.9 eV. This last one has two satellites at 5.7 eV and 3.5 eV. A maximum reflectivity value of 14.9% is obtained for a photon energy of 4.9 eV. In the considered energy domain, it remains above 8% and decreases with wavelength above 330 nm—energies below 3.75 eV. This is probably due to the capture of the free charge carriers originating from within the metallic film on the surface states of the substrate. Therefore, reflectivity data must be interpreted considering both photonic interactions with free electrons from the LaNi_5_ film, and with those captured in the metal-insulator interface. The observations for the 4.5 nm film are well correlated with Lorenz-Drude model for dielectrics, represented by the blue line in Figure 6a. The shape of the reflectance looks like at least a part of the absorptive, reflective, and probably transmissive regions are captured for this structure. It clearly acts like a dielectric in the depicted energy domain.

The highly visible peak-like structures observed for the 4.5 nm film can also be identified in the 22 nm thick film, as seen in Figure 6b. The slight variations that are present can be attributed to an increase in the concentration of the free charge carriers in the metallic thin film, which manifests itself in an increase of the reflectance with wavelength. Its general shape looks like at least a part of the reflective and transmissive region is still present in the UV-VIS domain, but with a much more pronounced metallic behavior.

For films thicker than 22 nm one can identify the poorly structured band signatures A, C, and H, around 2–3 eV, 5 eV, and 8 eV, using Figure 6c–e, respectively. These optical transitions could originate from the high density of states (DOS) *d*-type electron levels from deep inside the conduction band to energies above the Fermi energy. The reflectivity spectrum of the 36 nm film deposited on SiO_2_ substrate from Figure 6e exhibits better outlined transitions. One can identify a three-band structure—denoted here A, A′, and A′′—and the particularity G around 6.7 eV, which is barely visible elsewhere.

### 3.4. Refractive Index and Extinction Coefficient

**Figure 7:**
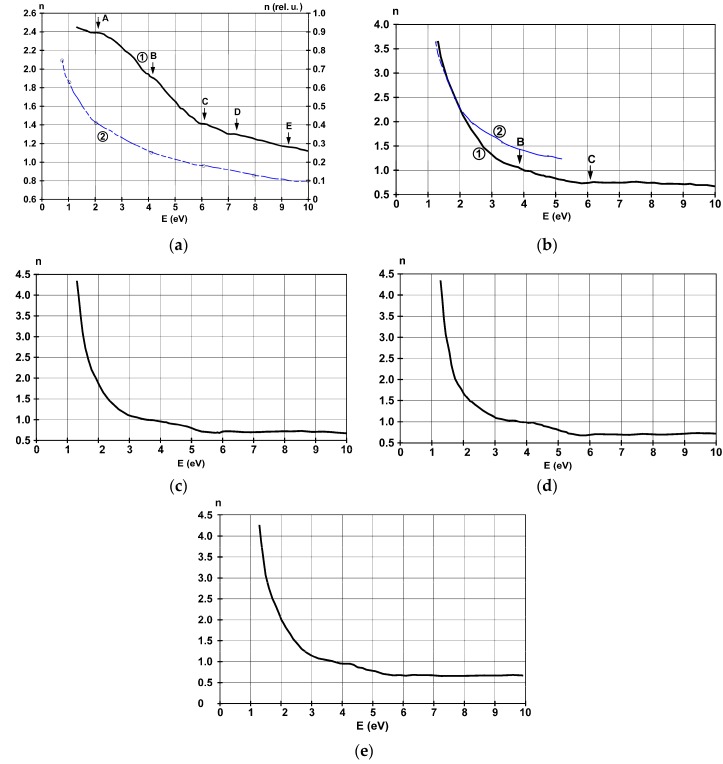
Spectral dispersion of the refractive index *n* for the films deposited on glass substrate with thicknesses of (**a**) 4.5 nm, (**b**) 22 nm, (**c**) 38 nm, (**d**) 42 nm, and (**e**) 36 nm on SiO_2_. The blue curves are computed from theory using the free electron approximation.

The spectral behavior of the refractive index is represented in Figure 7a–e, for the same films as in the previous subsection. This optical coefficient and the extinction coefficient were computed from the differential reflectance data discussed in the previous subsection using the Kramers-Krönig formalism. Because of the applied spectroscopic method, the reflections from the substrate-film interface and from the surface states are included, and the bare substrate’s reflectance is excluded from the experimental data. This is done to try to identify the electrons participating in the light absorption process, when the results computed from experimental data are compared to the ones resulting from Drude’s free electron theory.

The spectral dependence of *n*, for the thinnest film of 4.5 nm, found in Figure 7a, shows a poor concordance in shape and magnitude when compared to Drude’s free electron model, so that metallic behavior is not characteristic here. It exhibits increasing values with wavelength—decreasing tendency with energy—and weak characteristics at 2.2 eV, 4.1 eV, 6 eV, 7.3 eV, and 9.4 eV. Those located at 2.2 eV and 6 eV are also visible in the reflectance spectrum. In the high energy domain, the free electrons are the ones that contribute significantly to the refractive index. In the low energy domain, 1.5–5.5 eV, the shape of the behavior differs fundamentally from the theoretical one. The difference can be explained by the contribution of the electrons that are tightly bound in the surface states. The difference between curves (1) and (2) from Figure 7a could originate from the transitions of the *d*-electrons to the bands near the Fermi energy. Because the transitions denoted here as A and C were also identified in the reflectance spectrum as ones corresponding to maximum DOS energy bands, it is possible that the new ones denoted here D and E could have some similar origins.

Increasing the film thickness leads to an increase in free electronic contribution in the values of *n*, underlining the film’s more pronounced metallic behavior. The refractive index’s proportionality with the square root of the photon’s wavelength—observed from the approximate agreement in Figure 7b of the experimental curve and the theoretical one computed using the Drude formalism for free electrons—characterizes metals. The difference can be explained by the reflection on the film’s surfaces. The 42 nm thick film preserves the characteristic observed around 4 eV, explained by electronic transitions from the *3d* bands. Figure 7e proves that changing the substrate influences minimally this optical constant in the envisaged energy domain. Values of *n* lower than 1 can be explained by the fact that the velocity of light increases when it passes this medium, and the photon’s energy is around the electron plasma energy of the metallic film.

**Figure 8:**
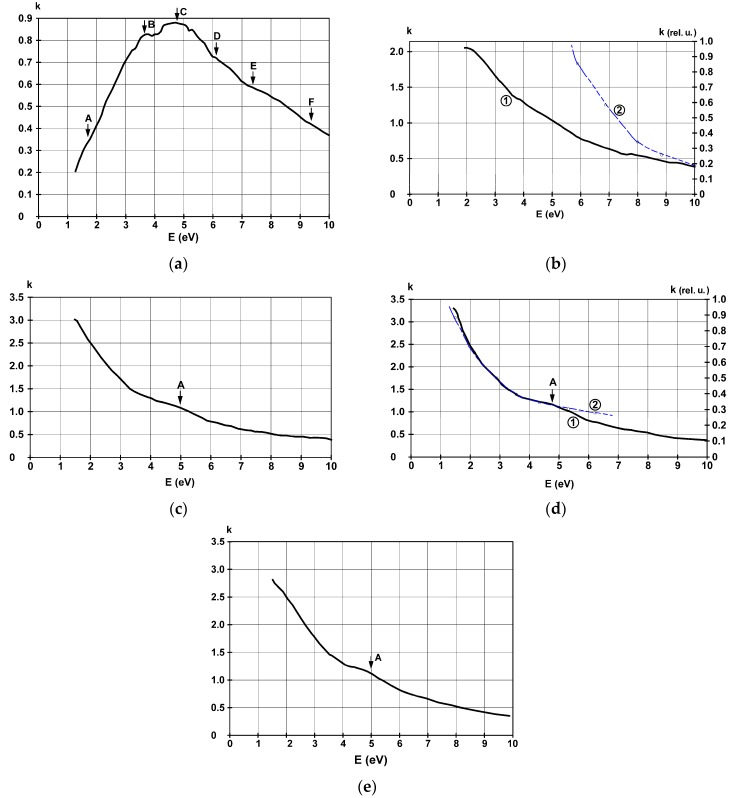
Spectral dispersion of the extinction coefficient *k* for the films deposited on glass substrate with thicknesses of (**a**) 4.5 nm, (**b**) 22 nm, (**c**) 38 nm, (**d**) 42 nm, and (**e**) 36 nm on SiO_2_. The blue curves are computed from theory using the free electron approximation.

The spectral dispersion of the extinction coefficient *k* for the same deposited films is represented in Figure 8.

The 4.5 nm film has the absorption characteristics of insular discontinuous metallic surfaces, and an electron confining energy barrier can be clearly identified with a dampening factor of at least a couple of electronvolts. The curve is characteristic of dielectrics, and it looks like at least a part of the absorptive and reflective region is captured in this energy domain. This is also in good concordance with observations made on the behavior of the reflectance that were drawn from Figure 5a. In the high energy domain, one can still find the characteristics at C (4.9 eV), D (6.1 eV), E (7.2 eV), and F (9.3 eV), which were discussed before, in this study. Decreasing values of optical absorption in the low energy domain is explained by a lower transition probability, because charge carriers are trapped in the metal-substrate interface. The characteristic marked here with A, can be due to the *s*-electrons originating from nickel and lanthanum’s conduction bands.

For the films with thicknesses higher or equal to 22 nm, optical absorption decreases with increasing energy, conforming to a generalized metallic behavior. Still, the determined values are much lower than those computed from the free electron approximation, so that this model cannot describe correctly the observed behavior. This can be explained by the fact that many electrons are still captured in the insularly deposited surface states of this intermetallic alloy, or at the substrate-metal contact region, which still occupies a relatively large area, and light can penetrate this deep to probe the strongly absorbing materials.

In the case of the 42 nm film, one can observe a rapid increase of the absorption in the NIR domain, up to 4.5 eV, while the contrary is true for UV-VIS energies. The coincidence of the blue line, computed using the free electron approximation, with the values computed from experimental reflectance data, confirms again the metallic behavior of this film, which can be considered thick when compared to light penetration distances.

Computing the skin depth—where the radiation is attenuated e ≈ 2.718 times—for a 500 nm wavelength incident electromagnetic radiation, using the classical Beer-Lambert law and the *k* values from Figure 8, Table 2 can be constructed.

When crossing the border between films under and over a couple of tens of nanometers, a quite big difference in radiation absorption properties can be observed. Above this value, although a monotone decrease is observed, the variation of skin depth is minimal, with a slope of approximately 3.5 × 10^–2^. These computed distances are consistent with light penetration figures in strongly absorbing metallic films [44]. The big value obtained in the thinnest film can be explained resorting to the model of insularly deposited films. Hence, the number of the free electrons decreases, because they are captured in the surface states, resulting in lower free electronic light absorption. This, corroborated with the high number of reflections on interfaces and surfaces, leads to longer paths that light can travel inside the probed medium. The above considerations underline the power of optical probing to study the structure of the valence band, especially when working with insular thin films of only a few nanometer thicknesses. However, of course, the same method fetches poor information from the thicker nanoscale films, because of free electron screening effects.

## 4. Conclusions

In this work we report successful deposition and study of electrical conductivity and optical properties of polycrystalline nanometer thickness LaNi_5_ films on mainly glass and a single SiO_2_ substrate. The thin films were prepared using pulsed laser deposition with short and modulated impulses and electro-optical tuning of the quality factor. The good linearity of layer thickness increase per laser impulse, over the whole deposition process, allowed us to prepare films with thicknesses raging from under 10 nm up to just below 200 nm. Thicknesses of the thinnest layers, below the limit of the interferometric optical microscopy measurement system, were determined by extrapolation, by counting the laser impulses. Beside thickness dependence on the number of laser impulses, another layer thickens modulation factor that was used was deposition geometry. XRD studies showed that good quality films were obtained. A crystallite size of 17 nm and a micro-strain below 0.5% was evaluated for the 68 nm film. Temperature dependent electrical conductivity measurements confirmed the insular deposition patterns of the thinnest films. These measurements also revealed gradually growing islands with laser impulse number, until a continuous film was obtained. The 180 nm thick film exhibited bulk-like metallic conductivity properties, with conductance decreasing with temperature. The inter-insular activation and hopping energy was evaluated for the 68 nm film, whose behavior was semiconductor-like due to this phenomenon. The interesting feature of the 110 nm film was that it equilibrated the metallic conduction behavior, characteristic to this alloy, with that of semiconductor-like behavior, modulated by the inter-insular hopping process, on a wide temperature domain. Absolute single-wavelength single-beam differential optical reflectance and transmittance studies, using a HeNe laser, showed that, from the point of view of optical features, bulk-like behavior is already regained at around a 70 nm film thickness. These measurements were further used to calibrate the single-beam differential reflectance spectroscopy determinations on a broad ultraviolet, visible, near- and mid-infrared (UV-VIS-NIR-MIR) domain. This broad domain, the applied interpolations, and the extrapolations down to 125 nm wavelength were necessary to complete the work using the Kramers-Krönig formalism, leading to the computation of the complex refractive index’s spectral behavior, with its real part the refractive index *n*, and its imaginary part, the extinction coefficient *k*. Reflectance data led us to the identification of the allowed electronic transitions near the Fermi energy, which appear tabulated in the text. Different substrates gave similar energy band structure determinations, but the film deposited on the SiO_2_ displayed them in a more eloquent fashion. Experimental data, and the results computed from it, were compared with Drude’s free electron theoretical model for every studied optical parameter and with the Lorentz-Drude model for the reflectance of the thinnest film. The observations what could be made this way led to the elucidation of the origin of the observed optical properties, which were signatures of electrons trapped at the metal-substrate interface, bound in the surface states, or of previously determined allowed interband transitions. During this process, comparisons with data obtained from referenced X-ray photoelectron spectroscopy (XPS) studies and theoretical first principle investigations of bulk LaNi_5_ were also used. This way, we could also support the choice of differential optical reflectance spectroscopy as a method for the investigation of the electron energy band structure by direct probing the allowed electronic transitions. We also proved that the thinnest layers are best suited to study electronic structures in metallic alloys. Clear signals were obtained because most of the free charge carriers were captured in the relatively high area of the surface states—both on the metal or metal-substrate interface—leaving free electronic energy states, where optical transitions occurred, from deeper energy bands or from near the Fermi energy. The reduction of free electron contribution to light attenuation and an increased number of interface reflections obtained for the films up to a couple of tens of nanometers also helped in probing a greater sample volume. Our study also correlated well with our previous conclusions drawn from the behavior of the CeNi_5_ nanoscale films. Therefore, this work brings fundamental knowledge on the band structure near the Fermi energy of the LaNi_5_ compound, obtained directly from the interpretation of experimental data, correlated with theoretical descriptions and referenced literature. Moreover, it also gives examples for different electrical conductivity behaviors that can be modulated by changing film thickness, implicitly by inter-insular hopping distances. This should help in better understanding this intermetallic compound’s behavior in its well-known everyday applications, and for the reduction of REE usage by using films composed of nanoscale particles.

## Figures and Tables

**Table 1:** Allowed interband transitions identified in the spectral behavior of reflectance.

Substrate	Film Thickness	Identified Allowed Interband Transitions (eV)							
		A	B	C	D	E	F	G	H
Glass	4.5 nm	2.2	3.4	4.9	5.7	6.1	6.5	6.8	8.2
	22 nm	2.3; 3	3.5	4.9	5.8	6	-	6.8	8.2
	38 nm	1.7; 2.9	-	4.7–5.2	-	-	-	-	8.1
	42 nm	1.6; 3	-	5.2	-	-	-	-	7.8–8.2
SiO_2_	36 nm	1.9; 2.4; 3	-	5.1	-	-	-	6.7	8.1

**Table 2:** Computed skin depth of a 500 nm incident electromagnetic radiation.

Film Thickness	Skin Depth
4.5 nm	60 nm
22 nm	9.8 nm
38 nm	9.5 nm
42 nm	9.1 nm
